# Exploring the Impact of Anxiety, Depression and Fatigue on Quality of Life Among Caregivers of Individuals With Mental Health Disorders

**DOI:** 10.7759/cureus.90243

**Published:** 2025-08-16

**Authors:** Maria Monachou, Marianna Mantzorou, Georgia Fasoi, Dimos Mastrogiannis, Theodoula Adamakidou, Alexandra Koreli, Ioannis Kalemikerakis, Paraskevi Apostolara, Marianna Drakopoulou, Georgia Gerogianni

**Affiliations:** 1 Department of Nursing, Postgraduate Program "Community and Public Health Nursing", University of West Attica, Athens, GRC

**Keywords:** anxiety, caregivers, depression, fatigue, psychiatric disorders, quality of life

## Abstract

Introduction: Caregivers for people diagnosed with mental health disorders can be more susceptible to experiencing increased levels of fatigue, anxiety, depression, and overall stress due to the demanding role of caregiving. This can significantly impact the physical and mental health of the care provider. The purpose of this study is to examine the impact of anxiety, depression, and fatigue on quality of life in caregivers of individuals with mental health disorders.

Methods: In this cross-sectional study, 112 caregivers of individuals with mental disorders completed the following scales: i) Hospital Anxiety and Depression Scale for the evaluation of anxiety and depression, ii) Kingston Caregiver Stress Scale for the evaluation of caregivers’ stress, iii) Fatigue Severity Scale for the evaluation of fatigue, iv) SF-12 Health Survey for the evaluation of quality of life, and v) A questionnaire about demographics. Variables were tested for normality using the Kolmogorov-Smirnov criterion. Quantitative variables were expressed as mean values (standard deviation) and as median (interquartile range). Qualitative variables were expressed as absolute (N) and relative frequencies (%). Spearman correlation coefficients (rho) were used to explore the association of two continuous variables. Multiple linear regression analyses were used with the SF-12 dimensions’ scores as dependent variables.

Results: Increased fatigue, higher levels of anxiety and depression, and increased overall stress were associated with poorer physical and mental health outcomes for caregivers of people with mental health disorders (p<0.001). An increase in fatigue was also associated with higher levels of anxiety, depression, and stress (p<0.001). Caregivers with a pre-existing health problem had poorer physical health outcomes (p<0.001). An extended duration of caregiving was associated with reduced physical health (p=0.017). Parental caregivers reported low mental health (p=0.005).

Conclusions: Caregiving was associated with increased fatigue, anxiety, depression and stress, leading to poorer physical and mental health outcomes for caregivers. Creating a coherent support framework that respects caregivers' needs and focuses on preventing burnout can play a key role in improving their well-being and quality of life, while strengthening the quality of care they provide.

## Introduction

The number of individuals with mental health disorders receiving care at home is steadily increasing. As a result, the family often takes on the role of being the primary source of support in the care of these individuals, with mental health disorders adding a significant burden on family caregivers [1].

Caregiving is a demanding responsibility that frequently causes significant psychological distress for the caregiver. Anxiety and depression are among the most common issues experienced. Caregivers often feel overwhelmed, experience reduced life satisfaction, and neglect their own health, which are strong indicators of depression [2]. They commonly face depression, anxiety, social isolation, and a decline in mental well-being due to caregiving burden [3].

Additionally, caregivers frequently experience sleep disturbances, feelings of abandonment, and a sense of hopelessness due to daily caregiving demands. Time constraints often limit their ability to maintain social connections, making loneliness and isolation common among those caring for individuals with schizophrenia or other mental illnesses. Emotional difficulties such as frustration and fear are also prevalent throughout the caregiving journey [2]. They often face difficulties with their occupational life, as they may reduce or adjust their working hours, have days off without receiving pay, resign from their jobs, or retire early in order to care for their patient [4].

As a result, the provision of care can impact many aspects of the caregivers’ lives. This can lead to mental, emotional, physical, and even financial difficulties. The responsibilities associated with caregiving can result in a decreased quality of life that may be characterized by social withdrawal, dissatisfaction, and physical health decline, as well as increasing vulnerability to depression, anxiety, and stress. In addition, caregiving can exacerbate or contribute to the development of physical conditions, including stomach ulcers, back pain, headaches, arthritis, and high blood pressure [5].

Although many studies have examined anxiety, depression, fatigue, or quality of life among caregivers, few studies have explored the interrelationship between those variables among caregivers of people with mental disorders in the Greek population. The aim of this study is the investigation of the impact of anxiety, depression and fatigue on quality of life among caregivers of individuals with mental health disorders.

## Materials and methods

This cross-sectional study aimed to investigate the impact of anxiety, depression and fatigue on the quality of life among caregivers of individuals with mental health disorders.

Study population

The study took place in Athens, Greece, involving a convenience sample of 112 caregivers of individuals with psychiatric disorders. The inclusion criteria for participants were to be over 18 years old, with a good ability of understanding, speaking, and reading in the Greek language. They should not have any psychiatric disorder or cognitive impairment, and should not misuse alcohol or substances. The exclusion criteria included inadequate language skills, age under 18 years old, cognitive decline, mental health conditions, and substance or alcohol misuse.

Caregivers who fulfilled the inclusion criteria were approached in the waiting area of a Mental Health Center in Athens, where they were verbally informed about the purpose and procedure of the study and confidentiality was guaranteed. Afterwards, they were given the questionnaires, in case they consented to participate. The study was conducted between September 2024 and February 2025. Questionnaires were distributed to 150 caregivers, of which 112 were returned, resulting in a response rate of 74.67%.

Data collection tools

In this study, Hospital Anxiety and Depression Scale (HADS), the Kingston Caregiver Stress Scale (KCSS), the Fatigue Severity Scale (FSS), and the SF-12 Health Survey (SF-12) were used to assess anxiety, depression, stress, fatigue, and quality of life in caregivers. Also, a scale about demographics was used.

Hospital Anxiety and Depression Scale (HADS)

The Hospital Anxiety and Depression Scale (HADS), developed by Zigmond and Snaith [6], was used to assess anxiety and depression levels. This tool includes 14 items, with seven questions measuring depression (items 2, 4, 6, 8, 10, 12, and 14) and the other seven assessing anxiety (items 1, 3, 5, 7, 9, 11, and 13). Respondents rate each question on a 4-point Likert scale ranging from 0 to 3. Scores for anxiety and depression are calculated separately by summing the respective items, with total scores ranging from 0 to 21. According to existing literature, scores of 0-7 indicate no anxiety or depression, 8-10 suggest moderate levels, and scores above 11 reflect high levels of anxiety or depression. The HADS has demonstrated strong reliability and validity in the Greek population, particularly among cancer patients [7].

Kingston Caregiver Stress Scale (KCSS)

The KCSS was used to assess stress experienced by family caregivers of individuals with dementia and was developed by Hopkins and Kilik. It is a self-administered questionnaire that allows caregivers to express their perceived stress while providing care to a family member. The scale consists of 10 questions that evaluate caregiver stress across three areas: caregiving (seven questions), family issues (two questions), and financial problems arising from caregiving (one question). Responses are rated on a scale from 1 to 5, where 1 indicates no stress and 5 indicates extreme stress. The total score ranges from 10 to 50. The scale has high reliability and validity in the Greek population (Cronbach's alpha = 0.85) [8].

Fatigue Severity Scale (FSS)

The Fatigue Severity Scale (FSS) is a questionnaire with nine items designed for self-assessment, developed by Krupp et al. [9], that measures the fatigue felt by respondents over the past two weeks. It was initially used to compare fatigue in individuals with systemic lupus erythematosus and multiple sclerosis [9]. The FSS has been translated into Greek and has high validity and reliability in the Greek population [10].

SF-12 Health Survey

The 12-item Health Survey (SF-12) was created as a shorter replacement for the SF-36, focusing on physical and mental health assessment [11]. It measures eight domains: physical functioning, role physical, role emotional, and mental health, using two questions each, while the remaining domains, bodily pain, general health, vitality, and social functioning are evaluated with one question each. All 12 items contribute to calculating the physical and mental component summary scores (PCS-12 and MCS-12) through a scoring method developed from data of a broad US population study. The SF-12 has proven to be a valid and effective tool to evaluate health-related quality of life in Greek people [12].

Statistical analysis

Variables were first tested for normality using the Kolmogorov-Smirnov criterion. Quantitative variables were expressed as mean values (standard deviation) and as median (interquartile range) (when the assumption of normality was not met). Qualitative variables were expressed as absolute (N) and relative frequencies (%). Spearman correlation coefficients (rho) were used to explore the association of two continuous variables. Multiple linear regression analyses, in a stepwise method, were used having the SF-12 dimensions’ scores as dependent variables (physical and mental health). The regression equation included terms for demographic characteristics, data on patient care, and the scales concerning fatigue, anxiety, depression and stress. Adjusted regression coefficients (β) with standard errors (SE) were computed from the results of the linear regression analyses. All reported p-values are two-tailed. Statistical significance was set at p<0.05 and analyses were conducted using IBM SPSS Statistics for Windows, Version 26 (Released 2019; IBM Corp., Armonk, New York, United States).

Ethical considerations

Ethical approval was received prior to data collection by Ethics Committee of the Attica Psychiatric Hospital (approval number 2/31-5-2024). The study was conducted in accordance with the Declaration of Helsinki (1989).

## Results

Demographic characteristics of caregivers

The sample consisted of 112 caregivers of individuals with mental disorders. The mean age of the participants was 60.1 years (SD = 13.9), while 72 (64.3%) were female. Regarding nationality, 95 (84.8%) were Greek. Regarding the relationship with the patient, 35 (31.2%) were children of the individuals they cared for, while 31 (27.7%) were siblings and 16 (14.3%) were parents. A total of 73 (65.2%) resided in the Attica region. In terms of academic education, 54 (47.2%) were secondary school graduates (middle/high school), 26 (23.2%) had completed primary education, and 32 (28.6%) had post-secondary or university education. More than half (64; 57.1%) were married, and 87 (77.7%) had children. Moreover, 64 (57.1%) were unemployed, 17 (15.2%) were public sector employees, 16 (14.3%) were employed in the private sector, and 15 (13.4%) were self-employed. Concerning their financial situation, 49 (43.8%) described it as moderate, 31 (27.7%) as good, and 25 (22.3%) as poor. A total of 64 (57.1%) of the caregivers reported having a health problem. Among them, 35 (54.7%) had hypertension, 30 (46.9%) musculoskeletal problems, 21 (32.8%) cardiovascular problems and 13 (20.3%) respiratory problems. Physical activity was reported by 34 (31.5%) of caregivers, with the majority of them (25; 73.7%) engaging in walking. Among those who exercised, 15 (44.1%) did so three times per week and 11 (32.4%) twice per week, with an average duration of 1.2 hours per session (SD = 0.5) (Table 1).

**Table 1:** Demographic characteristics of caregivers

		N	%
Gender	Male	40	35.7
	Female	72	64.3
Age. Mean (SD)		60.1 (13.9)	
Nationality	Greek	95	84.8
	Other	17	15.2
What is your relationship with the patient?	Parent (Father-Mother)	16	14.3
	Child (Son-Daughter)	35	31.2
	Siblings	31	27.7
	Other	30	26.8
Residence in the Attica region	No	39	34.8
	Yes	73	65.2
Educational level	Primary school	26	23.2
	Middle school - High school	54	47.2
	Post-secondary or higher education	32	28.6
Married/cohabitation	No	48	42.9
	Yes	64	57.1
Children	No	25	22.3
	Yes	87	77.7
Occupational status	Public sector employee	17	15.2
	Private sector employee	16	14.3
	Self-employed	15	13.4
	Unemployed	64	57.1
Financial status	Poor	25	22.3
	Moderate	49	43.8
	Good	31	27.7
	Very good	7	6.2
	Excellent	0	0.0
Do you suffer from a disease?	No	48	42.9
	Yes	64	57.1
Do you exercise?	No	74	66.1
	Yes	38	33.9

Caregiving responsibilities

A total of 50 (44.6%) of caregivers provided care all day, and the median duration of care was 120 months (IQR: 60-240 months). Additionally, 65 (58%) were the main caregivers, and 55 (49.1%) lived with the person they were caring for. Difficulties in providing care were reported by 54 (48.2%) in relation to medical services, 58 (51.8%) with social services, and 66 (58.9%) concerning financial benefits. Finally, 33 (29.5%) of the caregivers underwent psychotherapy, with a median duration of 24 months (IQR:12-36 months) (Table 2).

**Table 2:** Caregiving responsibilities

		N	%
How much do you help or care for the patient?	All day (24 hours)	50	44.6
	A few hours per day	27	24.1
	A few times per week	35	31.3
How long have you been helping or caring for the patient? (Months ), Median (IQR)		120 (60 – 240)	
Are you the main caregiver?	No	47	42.0
	Yes	65	58.0
Do you live with the patient?	No	57	50.9
	Yes	55	49.1
Do you face difficulties in caring for the person regarding medical services?	No	58	51.8
	Yes	54	48.2
Do you face difficulties in caring for the person regarding social services?	No	54	48.2
	Yes	58	51.8
Do you face difficulties in caring for the person regarding financial benefits?	No	46	41.1
	Yes	66	58.9
Are you undergoing psychotherapy?	No	79	70.5
	Yes	33	29.5
If yes, for how long? (months), Median (IQR)		24 (12 – 36)	

Descriptive statistics for SF-12, FSS, HADS and KCSS

The mean score on physical health dimension of the SF-12 scale was 42.1 points (SD = 12.0) and on the mental health dimension was 38.5 points (SD = 11.6). Higher values indicate better quality of life. The mean score on the FSS was 6.5 points (SD = 1.8), where higher values indicated greater fatigue.

On the HADS scale, the mean anxiety score was 10.9 points (SD = 5.8), and the mean depression score was 10.4 points (SD = 6.0). A higher score indicates greater anxiety or depression, respectively. Concerning the KCSS scale, the mean total stress score was 36.3 points (SD = 9.9), where higher values indicate greater stress (Table 3).

**Table 3:** Descriptive statistics for SF-12, FSS, HADS and KCSS SF-12: 12-item Health Survey; FSS: Fatigue Severity Scale; HADS: Hospital Anxiety and Depression Scale; KCSS: Kingston Caregiver Stress Scale

	Minimum	Maximum	Mean (SD)	Median (IQR)
Physical Component score (PCS)	21.8	63.8	42.1 (12.0)	-
Mental Component score (MCS)	17.8	62.1	38.5 (11.6)	-
Fatigue Severity Scale	9.0	63.0	37 (16.4)	38.5 (24 – 50)
Anxiety	0.0	21.0	10.9 (5.8)	11 (8 – 14)
Depression	0.0	21.0	10.4 (6.0)	10 (6 – 14)
Caregiver Stress scale (Kingston)	10.0	50.0	36.3 (9.9)	38.5 (29.5 – 45)

Spearman’s correlation coefficients for SF-12, FSS, HADS and KCSS

The physical health scale was significantly and negatively correlated with the fatigue scale (rho=-0.77; p<0.001), the anxiety (rho=-0.48; p<0.001) and depression dimensions (rho=-0.61; p<0.001) and the caregiver stress scale (rho=-0.38; p<0.001). This indicates that greater fatigue, higher anxiety, more intense depression, and increased overall stress were associated with poorer physical health.

Similar results were observed for the mental health scale, which was also significantly and negatively correlated with the fatigue scale (rho=-0.74; p<0.001), the anxiety (rho=-0.84; p<0.001) and depression dimensions (rho=-0.79; p<0.001) and the caregiver stress scale (rho=-0.75; p<0.001).

There was a significant and positive correlation among anxiety, depression, and caregiver stress (rho=0.84; p<0.001 and rho=0.82; p<0.001, respectively). Furthermore, fatigue was significantly and positively correlated with anxiety (rho=0.75; p<0.001), depression (rho=0.78; p<0.001), and caregiver’s stress (rho=0.68; p<0.001). Therefore, increased fatigue was associated with higher levels of anxiety, more severe depression, and elevated stress (Table 4).

**Table 4:** Spearman’s correlation coefficients between SF-12, FSS, HADS and KCSS *p<0.05, **p<0.01, ***p<0.001 SF-12: 12-item Health Survey; FSS: Fatigue Severity Scale; HADS: Hospital Anxiety and Depression Scale; KCSS: Kingston Caregiver Stress Scale

	Fatigue scale	Anxiety	Depression	Caregiver Stress Scale (Kingston)
Physical Component score (PCS)	-0.77***	-0.48***	-0.61***	-0.38***
Mental Component score (MCS)	-0.74***	-0.84***	-0.79***	-0.75***
Fatigue Severity Scale	1.00	0.75***	0.78***	0.68***
Anxiety			0.90***	0.84***
Depression				0.82***

Multiple linear regression analyses, in a stepwise method, with physical and mental health dimensions of the SF-12 scale as dependent variables

In order to find the factors that are independently associated with physical and mental health, multiple linear regression was performed. Demographic characteristics, data on patient care and the scales concerning fatigue, anxiety, depression and stress were used as independent variables. The presence of a health problem, fatigue, anxiety, duration of caregiving, relationship to the patient, and caregiver age were all associated with the caregiver’s physical health. Specifically, caregivers with a health problem had poorer physical health compared to those without one (β=-7.897, p<0.001). Greater fatigue (β=-0.457, p<0.001) and higher levels of anxiety (β=-0.449, p=0.005) were also related to poorer physical health.

Moreover, a longer duration of caregiving was associated with reduced physical health (β=-0.017, p=0.017), as well as greater age (β=-0.151, p=0.009). Among these factors, fatigue had the strongest impact on physical health, followed by the presence of a health problem. Increased fatigue (β=-0.191, p=0.002) and high levels of anxiety (β=-1.278, p<0.001) were significant burdening factors for mental health. In addition, caregivers who were parents of the patient reported worse mental health than those who were siblings (β=-5.471, p=0.005). Anxiety had the strongest impact on mental health, followed by fatigue (Table 5).

**Table 5:** Multiple linear regression analyses, in a stepwise method, with physical and mental health dimensions of the SF-12 scale as dependent variables. +regression coefficient ++standard error ±standardized regression coefficient

Physical Component Summary (PCS)	β+	SE++	b±	P
Do you suffer from a disease? (Yes vs No)	-7.897	1.453	-0.327	< .001>
Fatigue scale	-0.457	0.064	-0.622	< .001>
Anxiety	-0.449	0.155	-0.223	.005
How long have you been helping or caring for the patient? (months)	-0.017	0.007	-0.139	.017
Age	-0.151	0.057	-0.173	.009
Relationship with the patient: Parent vs Siblings	6.615	1.850	0.200	.001
Relationship with the patient: Child vs Siblings	3.525	1.820	0.136	.056
Relationship with the patient: Other vs Siblings	2.518	1.660	0.093	.133
Mental Component Summary (MCS)				
Fatigue scale	-0.191	0.061	-0.267	.002
Anxiety	-1.278	0.164	-0.646	< .001>
Relationship with the patient: Parent vs Siblings	-5.471	1.911	-0.169	.005
Relationship with the patient: Child vs Siblings	-.805	1.753	-0.032	.647
Relationship with the patient: Other vs Siblings	-2.412	1.608	-0.092	.137

## Discussion

This study showed that increased fatigue, high levels of anxiety and depression, and increased overall stress were associated with poorer physical and mental health outcomes for caregivers of people diagnosed with mental health issues (p<0.001). Demands of caregiving can significantly increase stress levels for caregivers and negatively impact their overall quality of life, including their mental well-being. It is estimated that about one-third to one-half of caregivers experience considerable psychological distress due to patients’ physical or cognitive impairment and the duration of the caregiving relationship, while patients may experience additional stress if their caregivers feel heavily burdened [13]. In a similar study, it was found that when patients exhibited high levels of anxiety and depression, their caregivers also experienced increased levels of anxiety and depression [14].

Psychosocial factors, such as feeling overwhelmed by caregiving responsibilities or facing tension in their relationships with patients, have been associated with a greater likelihood of individuals with dementia being placed in institutional care [13]. In a similar study involving 1,224 informal caregivers of individuals with mental illness, participants reported elevated levels of caregiver burden, depression, and anxiety, all of which were closely interrelated [15]. In another study with 131 caregivers of individuals with multiple sclerosis, caregivers’ fatigue, depression, and stress were found to be related to poor physical and mental health outcomes [16].

This study also found that increased fatigue was associated with high levels of anxiety, severe depression, and high levels of stress (p<0.001). It is important to take into consideration that fatigue and depression share several overlapping symptoms, including sleep disturbances, difficulty concentrating, and memory issues. These shared features may help explain why individuals experiencing fatigue are also more likely to report elevated levels of depression, anxiety, and stress, as these conditions frequently coexist [17]. A similar study involving 600 caregivers of psychiatric patients found that caregivers experienced significant physical, mental, and emotional fatigue, which was closely associated with higher levels of depression, anxiety, stress, and insomnia [18].

Additionally, the results of this study indicated that caregivers with a health problem had poorer physical health (p<0.001). Chronic physical conditions among caregivers are often worsened by factors such as ongoing stress, sleep disturbances, depression, unhealthy lifestyle habits, and financial strain. As a result, some caregivers may turn to smoking or alcohol as coping mechanisms, and may adopt a more sedentary lifestyle due to spending extended periods at home providing care. Moreover, caregivers often prioritize the needs of the person they care for over their own, leading to overlooking their own health concerns or considering them as less urgent. The demanding nature of caregiving can also leave little time or energy to seek medical attention for themselves [19].

Regarding physical health, family caregivers face a great possibility of developing cardiovascular disease [3]. In a similar study, caregivers’ perceived stress was significantly associated with poor self-rated health, which has been linked to a weakened immune system and is considered a strong predictor of mortality [20]. In another study, caregivers with multiple chronic health conditions reported high levels of perceived stress, particularly when caring for individuals with intensive needs. These caregivers often neglected their own physical and mental well-being. Research has shown a high prevalence of coexisting health issues among caregivers, including obesity, hypertension, depression, and sleep disturbances, which contribute to a decline in overall health [21]. In a similar study involving 285 informal caregivers of older adults, over half of them were found to have at least one chronic physical condition, while caregivers with one or more chronic health issues reported significantly lower physical quality of life [22].

Additionally, the findings of the present study showed that a long duration of caregiving was associated with reduced physical health (p=0.017). It is important to take into consideration that informal caregivers often face an increased risk of musculoskeletal pain and injuries, especially when engaging in demanding physical activities such as lifting or bathing the patient. These activities may lead to injuries and can elevate the likelihood of developing disorders such as osteoarthritis and other chronic health problems [5]. In a similar study, caregivers who engaged in intensive, long-term care were found to experience chronic stress, which can lead to a range of health issues, such as pain in muscles, hypertension, and weakened immunity [23]. 

The present study also showed that caregivers who were parents of the patient reported significantly low mental health (p=0.005). Since schizophrenia usually affects young individuals who have not yet started their own families, their primary caregivers are often their parents, and most commonly their mothers. Caring for a child with schizophrenia can substantially diminish caregivers’ quality of life and impose psychological, physical, and financial burdens, which can increase the risk of developing mood and anxiety disorders [24]. It is important to take into consideration that parent caregivers who support adolescents with mental disorders often carry a heavy emotional burden, including feelings of guilt, frustration, confusion, and helplessness, due to the unpredictable nature of their child’s symptoms and behavior. A significant part of this emotional strain derives from self-blame and guilt, frequently arising from the perception that greater efforts could have been made to prevent or address their child’s condition. This guilt deeply affects their well-being and can create doubt or insecurity within the parent-child connection. Consequently, these caregivers often place their child’s needs above their personal needs, exposing their own mental and emotional health at risk [25]. A similar study revealed that approximately 75% of caregivers experienced moderate to severe anxiety, along with moderate to severe caregiving burden. Caregivers’ concerns usually derive from the worsening condition of their children with mental health disorders [26]. Similarly, in a study with 414 caregivers of hemodialysis patients, parents or siblings presented significantly high levels of anxiety and depression due to limitations imposed by dialysis treatment in their daily life [27].

Moreover, the results of this study indicated that anxiety had the strongest effect on mental health, followed by fatigue. Long-term psychosocial stress can lead to burnout, which negatively affects daily life and is characterized by physical and mental exhaustion [28]. The burden of caregiving is caused by multiple responsibilities and often leads to difficulties in daily and leisure activities, social withdrawal, loss of hope, inadequate care for the patient, increased susceptibility to chronic illnesses, and, in some cases, patient abandonment [5]. Similarly, a study involving 84 family members caring for individuals with dementia, was found that high levels of anxiety among caregivers were associated with increased fatigue [29]. However, it has been found that caregivers who experience low care burden, receive adequate social support, maintain healthy marital relationships, understand the patient's condition well, apply effective coping strategies, and often exhibit a good quality of life [4].

The findings of the study show that caregivers of people with mental disorders can experience a complex and continuous emotional, physical, psychological and social burden, which affects their physical and mental well-being, significantly limiting their quality of life. In order to effectively relieve caregivers, it is necessary to design targeted interventions: psychoeducational programs, support groups, stress management techniques and temporary relief structures can strengthen their resilience and mental balance. At the same time, institutional recognition of the role of caregiver, financial support and access to specialized services are essential conditions for their sustainable support [30,31].

The creation of a coherent support framework, with respect to the needs of caregivers and with a focus on preventing burnout, can contribute not only to improving their own lives, but also to strengthening the care they provide. Ultimately, caring for caregivers is an integral part of patient care. This means that the community healthcare professionals systematically consider caregivers’ physical and psychological needs. Routine assessment of caregiver burden must be incorporated into clinical practice enabling timely identification and appropriate intervention. A range of evidence-based strategies have been proposed aiming to enhance the caregiver resilience, such as psychoeducational programs that improve the caregiver knowledge, strengthen their coping mechanisms and decision-making skills; self-care practices such as physical exercise, spiritual and mindfulness interventions can mitigate stress and foster quality of life. Additionally, psychotherapeutic and counseling interventions at the individual or group level further support psychological well-being. Moreover, community healthcare professionals play a pivotal role in bridging the gap between the caregiver and the healthcare system. They are responsible for proposing and referring caregivers to community resources, including respite care, financial and insurance support services, helplines, and national organizations. They also serve as advocates for caregivers’ rights and needs within the healthcare system, ensuring a holistic, inclusive, and coordinated approach to caregivers’ health [1,32]. 

The findings of this study have some limitations. The study was conducted at a single mental health center located in the prefecture of Attica, rather than at a national level, a fact that limits the generalizability of the findings to broader populations. The cross-sectional design restricts the ability to establish causal relationships, and the use of convenience sampling may introduce selection bias. Moreover, the over-representation of women and individuals of Greek nationality among participants may limit the applicability of the findings to male caregivers or those from different cultural backgrounds, who may face unique challenges or hold different perspectives regarding caregiving. In addition, the use of self-reported questionnaires presents a risk of response bias, which may influence the accuracy of the data. Another limitation is the lack of assessment of patients’ clinical status or the psychiatric symptom severity, which may have influenced caregiver burden and limited the ability to explore potential correlations between patient condition and caregiver well-being. Another limitation of the study is the fact that the caregivers’ health issues cannot be solely attributed to the burden of care, as other factors, such as genetic factors or even their own aging process, predispose them to musculoskeletal disorders, hypertension, and other health problems. Therefore, future studies using more diverse samples and longitudinal or mixed-methods designs are recommended. Despite any limitations, the results obtained from similar research are regarded as specifically useful for understanding how the burden of caring for people with mental health issues affects the mental health of caregivers and for carrying out additional research focused on creating sustainable public health interventions.

## Conclusions

Providing care is associated to high levels of fatigue, anxiety, depression, and stress. This can contribute to declines in both physical and mental health. Extended caregiving periods and existing health issues are associated with poorer physical health, while parental caregivers experience reduced mental well-being. Consequently, adopting extensive support interventions such as counseling, stress reduction initiatives, and improved healthcare access, combined with personalized mental health treatments, is vital to address the complex difficulties caregivers encounter and to enhance their physical and emotional health.
